# Supplementary material for: Synthesis of Zn2+-Pre-Intercalated V2O5·nH2O/rGO Composite with Boosted Electrochemical Properties for Aqueous Zn-Ion Batteries
Source: Molecules. 2022 Aug 24;27(17):5387. doi: 10.3390/molecules27175387 (PMC9457629; doi:10.3390/molecules27175387)
Supplement: Supplementary file 1 [file molecules-27-05387-s001.zip › molecules-1846496-supplementary.pdf]

Supplementary Material

for

Synthesis of Zn^{2+}-pre-intercalated $V_2O_5·nH_2O$/rGO composite with boosted electrochemical properties for aqueous Zn-ion batteries

Yanzhi Fan[1], Xiaomeng Yu[2], Ziyi Feng[2], Mingjie Hu[3]* and Yifu Zhang[2]*

[1] Beijing Aerospace Intelligent Construction Co., LTD, Beijing, 102600, PR China
[2] State Key Laboratory of Fine Chemicals, School of Chemical Engineering, Dalian University of Technology, Dalian, 116024, PR China
[3] Hubei Key Laboratory of Advanced Aerospace Propulsion Technology, Hubei Military-Civilian Integration and Co-Innovation Center of Aerospace Propulsion and Materials Technology, Wuhan 430040, China
* Correspondence: humingjie1987@whu.edu.cn (M.H.); yfzhang@dlut.edu.cn (Y.Z.)

Materials

Graphite sheet (99.95%) was purchased from Shanghai Aladdin Industrial Co., Ltd., Shanghai, China. Sulfuric acid (98 wt%), hydrochloric acid, sodium nitrate (99%), high manganese potassium (95%), hydrogen peroxide (H_2O_2, 30%), ammonium metavanadate (99 %), zinc sulfate ($ZnSO_4$, 99%) and acetic acid glacial (99.5%) were ordered from Siopharm Chemical Reagent Co., Ltd., Tianjing, China. All chemicals with analytical grade were directly used without any further purifications.

Materials Characterizations

X-ray diffraction (XRD) patterns were measured using Panalytical X' Pert powder diffractometer at 40 kV and 40 mA with Ni-filtered Cu Kα radiation. The functional groups' stretching and bending information of ZnVOH/rGO was characterized by Fourier transform infrared spectroscopy (FTIR) and recorded on a Nicolet 6700 spectrometer from 4000 to 400 cm^{-1} with a resolution of 4 cm^{-1}. Thermo Scientific spectrometer was used to obtain the chemical bond information by Raman spectrum, with a 532 nm-excitation line (DXR Microscope03030429). Energy-dispersive X-ray spectrometer (EDS) elemental mapping was obtained by a scanning electron microscope (SEM, QUANTA450). Inductive coupled plasma emission spectrometer (ICP) was carried out on Solar969MK11. The morphology and size of ZnVOH/rGO were displayed by field emission scanning electron microscopy (FE-SEM, NOVA NanoSEM 450, FEI) and transmission electron microscopy (TEM, FEITecnai F30, FEI). Energy-dispersive X-ray spectrometer (EDS) and elemental mapping were obtained by a scanning electron microscope (SEM, QUANTA450).

Electrochemical measurements

The electrochemical properties of the ZnVOH/rGO cathode were evaluated using CR2032 coin-type cells, which were assembled by the as-made ZnVOH/rGO cathode, Zn anode and the 3M $Zn(CF_3SO_3)_2$ electrolyte. A glass fiber filter was used as the separator. The potential range of the Zn//ZnVOH/rGO battery was chosen to be 0.2~1.4 V (vs. Zn^{2+}/Zn). The cyclic voltammetry (CV) and electrochemical impedance spectroscopy (EIS) of the Zn//ZnVOH/rGO battery were carried out using the CHI-660D electrochemical working station. The galvanostatic charge-discharge (GCD) and galvanostatic/intermittent titration technique (GITT) of the Zn//ZnVOH/rGO battery were conducted in a multichannel battery testing system (LAND CT201A). The electrochemical characterizations were tested at room temperature.

Figure S1

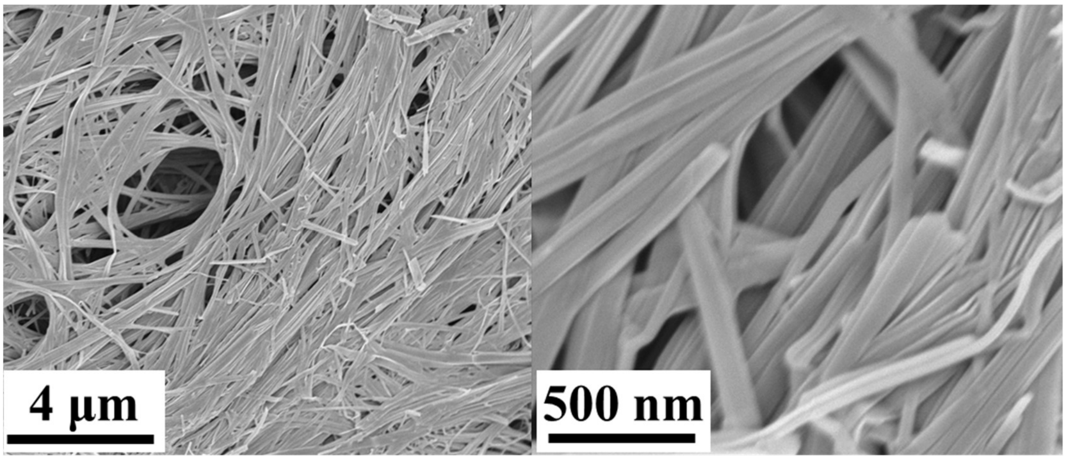

Figure S1. SEM images of ZnVOH.

Figure S2

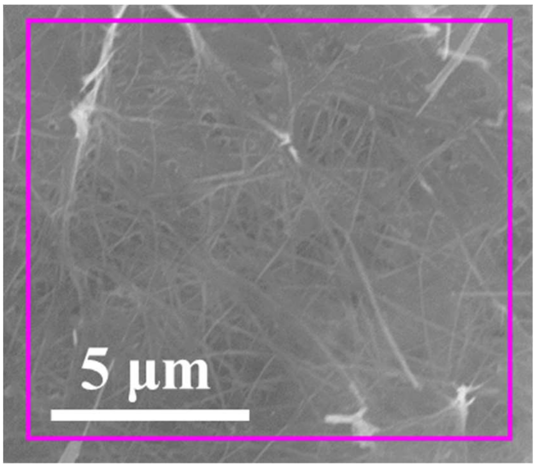

Figure S2. A SEM image of ZnVOH/rGO for collecting elemental mapping images.

Figure S3

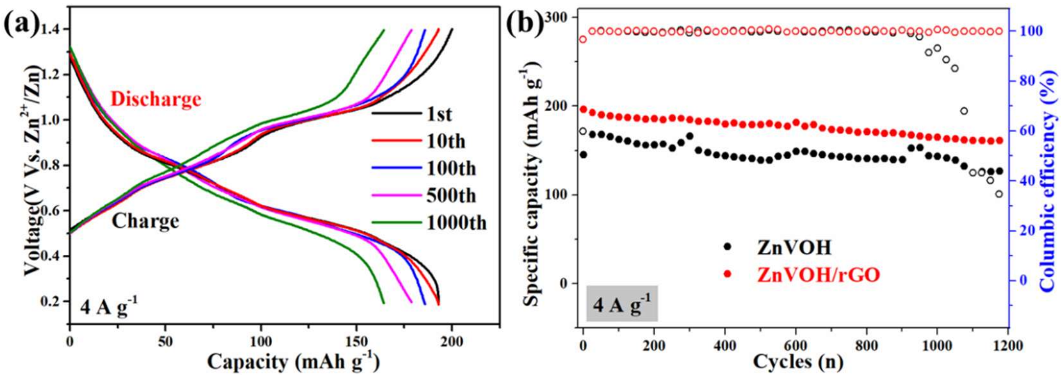

Figure S3. (a) Cycle performance and (b) GCD curves at 4 A·g^{-1} of ZnVOH and ZnVOH/rGO.

Figure S3 represents GCD curves for different cycles and cycle performance of ZnVOH at 4 A·g^{-1}. There are two voltage plateaus in these GCD curves, which well coincides to the CV curves with a multistep intercalation process. At 4 A·g^{-1} (Figure S3b), ZnVOH/rGO exhibits a capacity of 196 mA h g^{-1} and after 1200 cycles this value keeps 161 mA h g^{-1}, whereas, ZnVOH shows a capacity of 145 mA h g^{-1} and keeps 126 mA h g^{-1} after 1200 cycles.

Table S1

Table S1. Atomic ratio of Zn and V in ZnVOH by ICP analysis.

**Element**	**Atomic tatio**
**Zn:V**	**0.109:1**

Table S2

Table S2. Atomic ratio of Zn and V in ZnVOH/rGO by ICP analysis.

**Element**	**Atomic tatio**
**Zn:V**	**0.107:1**

Table S3

Table S3. Comparison of the specific capacities between the previously reported cathode materials for AZIBs and this work.

Cathode Materials	Electrochemical Performance	Ref.
**ZnVOH/rGO**	**325 mAh·g^{-1} at 0.1 A·g^{-1}**	**This work**
VO_2(B) nanobelts	274 mAh g^{-1} at 0.1 A g^{-1}	[51]
RGO/VO_2 composite	276 mAh g^{-1} at 0.1 A g^{-1}	[52]
V_2O_5 nanofibers	319 mAh g^{-1} at 0.02 A g^{-1}	[53]
$Ba_{1.2}V_6O_{16}\cdot 3H_2O$	321.2,277.3, 247.4, 222.8, 198.2,168.1, 150.7, 129.8, and 108.8 mA h g^{-1} at 0.1, 0.2, 0.3, 0.5, 1, 2, 3, 5, and 10 A g^{-1}	[54]
$FeVO_4\cdot nH_2O$@rGO	100 mA h g^{-1} at 1.0 A g^{-1}	[55]
$Cu_3(OH)_2V_2O_7\cdot 2H_2O$	216, 159, 148, 133, 127, and 105 mA h g^{-1} at 0.1, 0.3, 0.5, 0.8, 1.0, and 2.0 A g^{-1}	[56]
$K_{0.25}V_2O_5$	205,163 and 91 mA h g^{-1} at 1, 2, 5 A g^{-1}	[57]
LiV_3O_8	256, 311, 172, 148, and 47 mAh g^{-1} at 0.016, 0.066, 0.133, 0.266 and 1.066 A g^{-1}, respectively.	[58]
$Na_{1.1}V_3O_{7.9}$ nanoribbons/graphene	191 mAh g^{-1} at 0.05 A g^{-1}	[59]
$Na_5V_{12}O_{32}$ ($Na_{1.25}V_3O_8$)	281 mAh g^{-1} at 0.5 A g^{-1}	[60]
$Zn_3V_2O_7(OH)_2\cdot 2H_2O$	200, 122, 84 and 54 mAh g^{-1} at 0.05, 0.5, 1 and 3 A g^{-1}, respectively	[61]
$Zn_2(OH)VO_4$	204, 160 and 101 mAh g^{-1} at 0.5 C, 10 C and 50 C, respectively. (1 C= 200 mA g^{-1})	[62]
